# Contamination of *Proteus mirabilis* harbouring various clinically important antimicrobial resistance genes in retail meat and aquatic products from food markets in China

**DOI:** 10.3389/fmicb.2022.1086800

**Published:** 2022-12-16

**Authors:** Wan-Qing Ma, Ying-Yue Han, Lin Zhou, Wen-Qi Peng, Ling-Ya Mao, Xue Yang, Qin Wang, Tie-Jun Zhang, Hong-Ning Wang, Chang-Wei Lei

**Affiliations:** Key Laboratory of Bio-Resource and Eco-Environment of Ministry of Education, College of Life Sciences, Sichuan University, Animal Disease Prevention and Food Safety Key Laboratory of Sichuan Province, Chengdu, China

**Keywords:** *Proteus mirabilis*, food markets, whole genome sequencing, multidrug resistance, NDM, mobile genetic elements

## Abstract

*Proteus mirabilis* is an opportunistic pathogen frequently associated with nosocomial infection and food poisoning cases. Contamination of *P. mirabilis* in retail meat products may be important transmission routes for human infection with *P. mirabilis*. In this study a total of 89 *P. mirabilis* strains were isolated from 347 samples in 14 food markets in China and subjected to whole-genome sequencing. Phylogenetic analysis showed that all 89 strains were divided into 81 different clones (SNPs >5), indicating high genetic diversity of *P. mirabilis* in food markets. Antimicrobial susceptibility testing showed that 81 (91.01%) strains displayed multidrug resistance profiles. Seventy-three different resistance genes (or variants) were found, including various clinically important antimicrobial resistance genes *aac(6′)-Ib-cr* (77.53%), *bla*_CTX-M_ (39.33%), *fosA3* (30.34%), as well as multiresistance gene *cfr* (4.50%), tigecycline resistance gene cluster *tmexCD3-toprJ1* (4.50%) and carbapenemase gene *bla*_NDM-1_ (1.12%). Diverse genetic elements including Tn*7* transposon, plasmid, SXT/R391 integrative conjugative element were associated with the horizontal transfer of *cfr*. *tmexCD3-toprJ1* and *bla*_NDM-1_ were located on ICE*Pmi*ChnJZ26 and *Salmonella* genomic island 1, respectively. Our study emphasized high contamination of *P. mirabilis* harbouring various clinically important antimicrobial resistance genes in retail meat and aquatic products, which might be an important issue in terms of food safety and human health.

## Introduction

Food contamination caused by foodborne and opportunistic pathogenic bacteria that are leading causes of foodborne illnesses and deaths is a serious threat to global public health (Tropea, 2022). *Proteus mirabilis* is a species of Gram-negative bacteria belonging to genus *Proteus* of Enterobacterales (Janda and Abbott, 2021), and is widely distributed in the natural environment and intestines of humans and animals. *P. mirabilis* is one of the most frequently opportunistic pathogen causing nosocomial infection, especially causing urinary tract infection (Schaffer and Pearson, 2015). In addition, *P. mirabilis* infections can also cause bacteremia and the formation of urinary stones. It is worth noting that some food poisoning cases associated with *P. mirabilis* had been reported in China, as well as other countries (Gong et al., 2019). Given that *P. mirabilis* is a resident in the intestinal tract of chickens and swines, it can potentially be present in retail meat products due to fecal contamination at the slaughtering stage. Manipulation and consumption of retail meat products may be important transmission routes for human infection with *P. mirabilis*. Contamination of *P. mirabilis* in retail meat products has become an important issue for food safety and human health.

The wide use of antimicrobials for the treatment of bacterial infections in humans and food-producing animals has led to widespread dissemination of antimicrobial resistance genes. Emergence and spread of multidrug resistant (MDR) *P. mirabilis* isolates, including those producing extended-spectrum beta-lactamases (ESBLs), AmpC cephalosporinases and carbapenemases, raised concerns in the past few years (Girlich et al., 2020). *P. mirabilis* is intrinsically resistant to nitrofurantoin, polymyxin and tigecycline, which brings a serious challenge for the treatment of *P. mirabilis* infections. Many mobile genetic elements, including plasmids, integrons, insertion sequences and transposons (Partridge et al., 2018), as well as SXT/R391 integrative conjugative elements (ICEs) and genomic islands (such as *Salmonella*/*Proteus* genomic islands, SGI/PGI), are found to mediate horizontal transfer of various antimicrobial resistance genes in *P. mirabilis* (Cummins et al., 2020; He et al., 2021). For example, the New Delhi metallo-β-lactamase gene *bla*_NDM-1_ was sporadically found in *P. mirabilis*, and was located on plasmids (Bitar et al., 2020), PGI1 and SGI1 (Girlich et al., 2015; Yang et al., 2021). The multiresistance gene *cfr* that encodes an RNA methyltransferase conferring resistance to phenicols, lincosamides, oxazolidinones, pleuromutilins and streptogramin A (Shen et al., 2013), as well as the plasmid-encoding RND efflux pump gene cluster *tmexCD3-toprJ3* that reduces the sensitivity of multiple antimicrobials including tigecycline (Lv et al., 2020), were found to be located on SXT/R391 ICEs (Wang et al., 2021b; Song et al., 2022). These findings indicate that *P. mirabilis* is an important reservoir of clinically important antimicrobial resistance genes and mobile genetic elements that have the potential to spread to other pathogenic bacteria in Enterobacterales.

Traditionally, pulsed field gel electrophoresis was used for molecular typing, and there is no standard multilocus sequence typing for *P. mirabilis*. With the development of the next-generation sequencing (NGS) technology, phylogenetic analysis based on the single nucleotide polymorphisms (SNPs) in core genomes provides higher distinguishability for tracing the transmission of associated pathogens (Schurch et al., 2018). In China, food markets that sell retail meat (especially chicken and pork meat) and aquatic products are widely distributed in communities. However, the data of the contamination of *P. mirabilis* in those products is limited. The aims of the present study were to (i) investigate the prevalence of *P. mirabilis* in retail meat and aquatic products in food markets using NGS, (ii) detect antimicrobial resistance phenotypes and genotypes, and (iii) determine the mobile genetic elements associated with the clinically important antimicrobial resistance genes.

## Results

### Prevalence of *Proteus mirabilis* in retail meat and aquatic products from food markets

A total of 89 *P. mirabilis* strains (62 from chicken meat, 20 from pork meat and 7 from aquatic products) were isolated from 347 samples, and the overall isolation rate of *P. mirabilis* was 25.65% (89/347). The chicken meat samples had the highest isolation rate, which reached 54.39% (62/114). The isolation rates of *P. mirabilis* in pork and aquatic products were low, which were 14.18% (20/141) and 7.61% (7/92), respectively. In 14 food markets, the isolation rates of *P. mirabilis* varied from 4.76% (1/21) to 53.33% (8/15) (Table 1). The detailed information of the 89 *P. mirabilis* strains reported in this study is listed in Supplementary Table S1.

**Table 1:** Isolation rates of *P. mirabilis* in 14 food markets.

Food markets	Isolation rates of *P. mirabilis* (Numbers of strains/samples)^a^			
	Chicken meat	Pork meat	Aquatic products	Total
F1	57.14% (4/7)	0% (0/14)	25% (1/4)	20% (5/25)
F2	60% (3/5)	25% (1/4)	0% (0/2)	36.36% (4/11)
F3	60% (9/15)	14.29% (2/14)	8.33% (1/12)	29.27% (12/41)
F4	100% (5/5)	50% (3/6)	0% (0/4)	53.33% (8/15)
F5	55.56% (5/9)	10% (1/10)	–	31.58% (6/19)
F6	83.33% (5/6)	7.14% (1/14)	0% (0/10)	20% (6/30)
F7	75% (6/8)	25% (3/12)	75% (3/4)	50% (12/24)
F8	50% (1/2)	16.67% (1/6)	-	25% (2/8)
F9	-	-	12.5% (2/16)	12.5% (2/16)
F10	75% (3/4)	0% (0/4)	0% (0/2)	30% (3/10)
F11	77.78% (7/9)	12.5% (1/8)	0% (0/8)	32% (8/25)
F12	0% (0/12)	11.11% (1/9)	–	4.76% (1/21)
F13	30% (6/20)	5% (1/20)	0% (0/16)	12.5% (7/56)
F14	66.67% (8/12)	25% (5/20)	0% (0/14)	28.26% (13/46)
Total	54.39% (62/114)	14.18% (20/141)	7.61% (7/92)	25.65% (89/347)

^a^ –, none products for sale.

### Phylogenetic analysis of *Proteus mirabilis* strains

**Figure 1:**
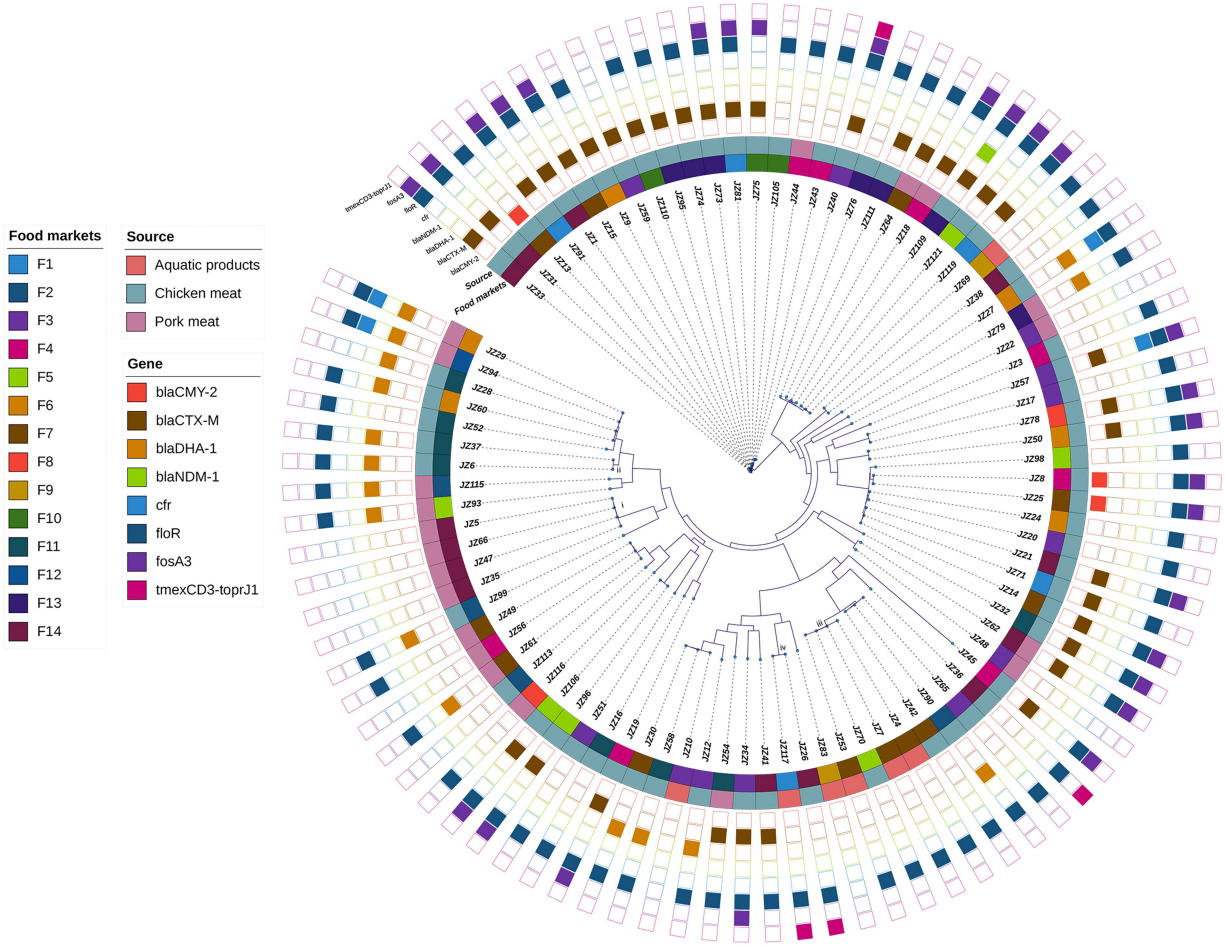
Phylogenetic analysis of 89 *P. mirabilis* strains. The food markets, sources and the presence of some clinically important antimicrobial resistance genes are indicated by different colours. i–iv represent four clonal transmission events (SNPs ≤5 among strains in the same clade).

The mean of the draft genomes of 89 *P. mirabilis* strains was 4.00 Mb (from 3.79 to 4.32 Mb) in size with average GC content of 38.8%. The draft genomes consisted of an average of 95 (from 49 to 260) contigs above 200 bp (Supplementary Table S1). A total of 83,751 SNPs were identified in the genomes of the 89 *P. mirabilis* strains, and the phylogenetic tree based on the SNPs was conducted (Figure 1). All 89 strains were divided into 81 different clones (SNPs >5), indicating high genetic diversity of *P. mirabilis* in food markets. However, four clonal transmission events were also found: (i) four clonal strains JZ5, JZ35, JZ47 and JZ66 (0–1 SNPs) were isolated from pork meat samples in food market F14; (ii) two strains JZ6 and JZ37 (4 SNPs) that harboured *bla*_DHA-1_ came from chicken meat samples in F11; (iii) four strains (JZ4, JZ7, JZ53 and JZ70) were clonally related (0–1 SNPs), in which three from aquatic products in F7, but JZ70 from chicken meat in F5; and (iv) strains JZ26 and JZ117 (5 SNPs) respectively came from chicken meat in F14 and aquatic product in F1, both of which harboured tigecycline resistance gene cluster *tmexCD3-toprJ1*.

### Antimicrobial resistance profiles of *Proteus mirabilis*

Antimicrobial susceptibility testing showed that the *P. mirabilis* strains exhibited high resistance rate to SXT (94.38%), STR (93.26%), CHL (86.52%), FFC (83.15%) and AMP (83.15%), and low resistance rate to CAZ (16.85%), IPM (13.48%) and AMK (5.62%). The resistance percentage to other agents was as follows: NAL (79.78%), NOR (69.66%), GEN (49.44%), CRO (49.44%), CIP (42.70%), FOS (30.34%), FOX (26.97%) and AMC (22.47%). It’s worth noting that 81 (91.01%) strains displayed MDR profiles.

**Figure 2:**
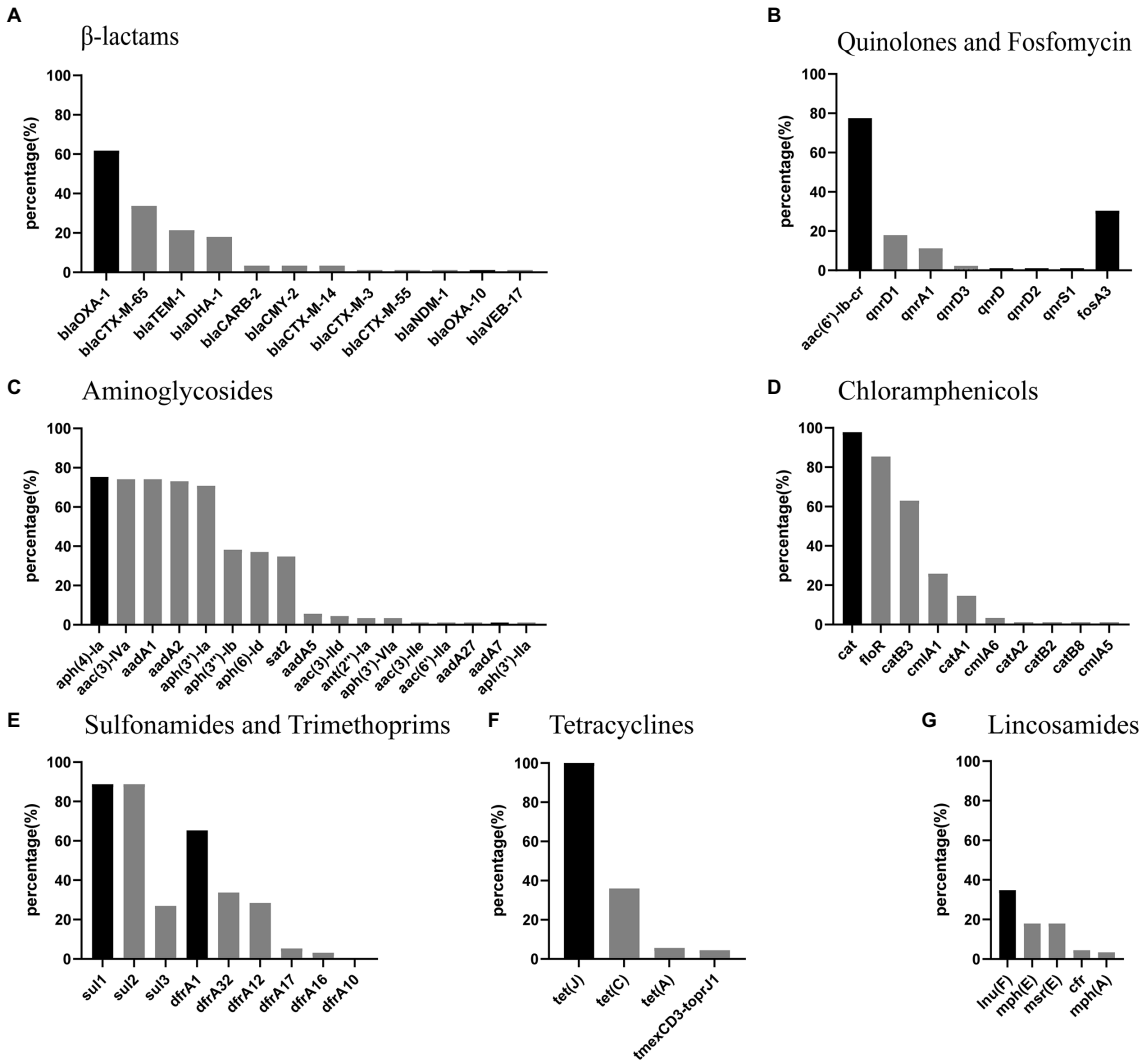
Percentage of antimicrobial resistance genes in *P.mirabilis* strains from food markets. **(A-G)** the most prevalent resistance genes for each class of antimicrobials are in deep black. Note: *cfr* is a multiresistance gene conferring resistance to lincosamides, phenicols, oxazolidinones, pleuromutilins, and streptogramin A.

A total of 73 different resistance genes (or variants) were found in the 89 *P. mirabilis* strains (Supplementary Table S1). The prevalence of β-Lactam, quinolone, fosfomycin, aminoglycoside, chloramphenicol, sulfonamide, trimethoprim, tetracycline and lincosamide resistance genes were shown in Figure 2. Intrinsical resistance genes *tet*(J) (tetracycline) and *cat* (chloramphenicol) were detected in 89 (100%) and 87 (97.75%) strains, respectively. *bla*_OXA-1_ was the most prevalent β-lactamase genes, and found in 55 (61.80%) strains. Thirty-five strains carried ESBLs gene *bla*_CTX-M_, including 30 *bla*_CTX-M-65_, 3 *bla*_CTX-M-14_, 1 *bla*_CTX-M-3_ and 1 *bla*_CTX-M-55_. AmpC β-lactamases genes *bla*_DHA-1_ and *bla*_CMY-2_ were found in 16 and 3 strains, respectively. More importantly, one strain (JZ109) carried carbapenemase gene *bla*_NDM-1_. Quinolone resistance gene *aac(6′)-Ib-cr* was found in 69 (77.53%) strains. Nineteen strains carried plasmid-mediated quinolone resistance gene *qnrD*, including 16 *qnrD1*, 1 *qnrD2* and 2 *qnrD3*. Fosfomycin resistance gene *fosA3* was detected in 27 (30.34%) strains. The prevalence of other resistance genes above 60% was as follows: aminoglycoside resistance genes *aph(4)-Ia* (75.28%), *aac(3)-IVa* (74.16%), *aadA1* (74.16%), *aadA2* (73.03%) and *aph(3′)-Ia* (70.79%); chloramphenicol resistance genes *floR* (85.39%) and *catB3* (62.92%); sulfonamide resistance genes *sul1* (88.76%) and *sul2* (88.76%). It’s worth noting that multiresistance gene *cfr* and tigecycline resistance gene cluster *tmexCD3-toprJ1* were found in 4 and 4 strains, respectively.

### Genetic environments and transferability of clinically important antimicrobial resistance genes

**Figure 3:**
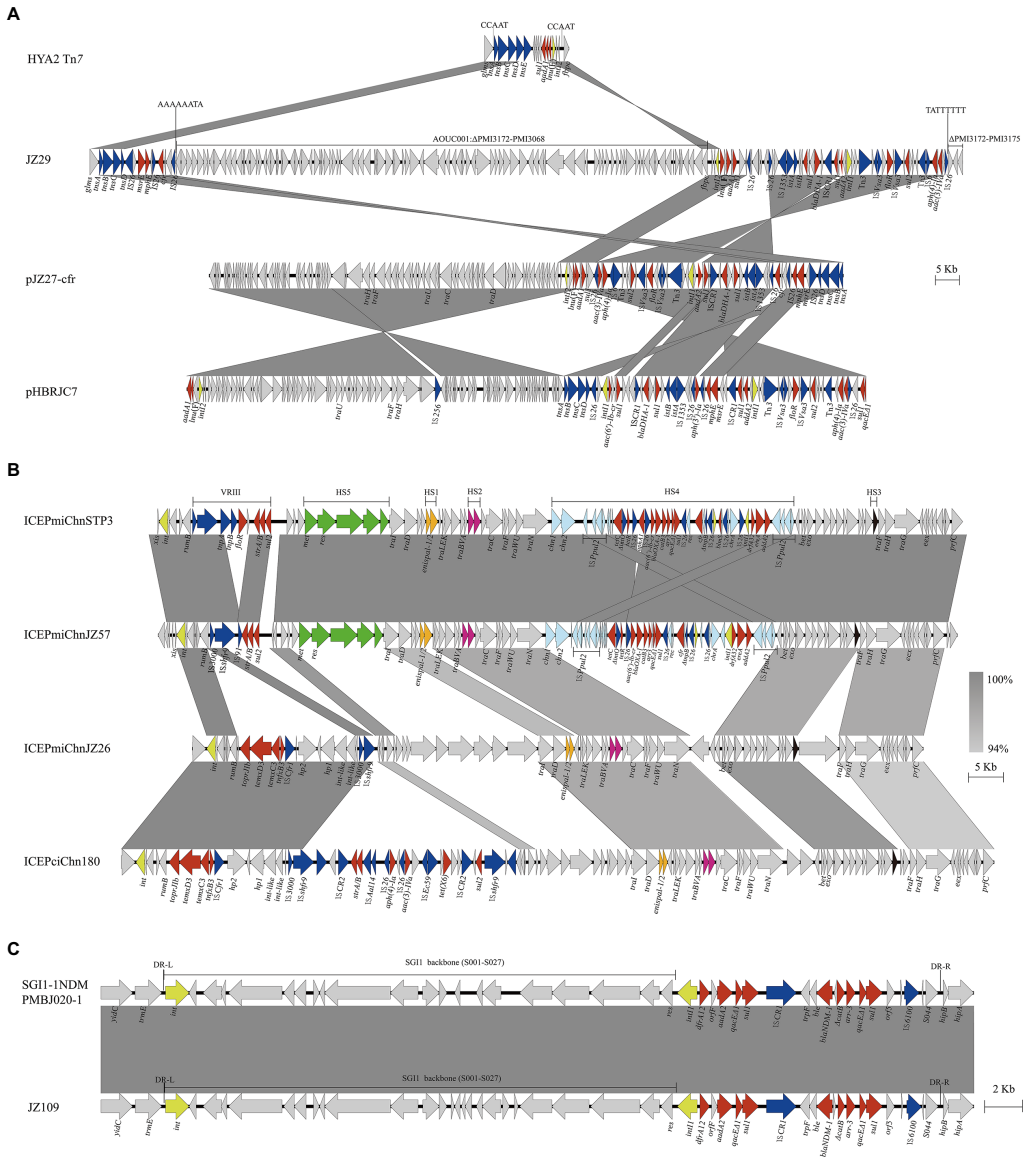
Genetic environments of clinically important antimicrobial resistance genes *cfr*, *tmexCD3-toprJ1* and *bla*_NDM-1_. These genes were associated with Tn*7* transposons in chromosome or plasmid **(A)**, SXT/R391 ICEs **(B)**, and SGI1 **(C)**. Genes and ORFs are shown as arrows, and their orientations of transcription are indicated by the arrowheads. Shared regions of >99% nucleotide sequence identity are shaded in gray. Integrase genes, resistance genes and transposase genes are in yellow, red, and blue, respectively. HS1-HS5 and VRIII represent the hot spots 1–5 and variable region III in SXT/R391 ICE. DR-L and DR-R represent the 18-bp direct repeats at the ends of SGI1.

Genetic environments of clinically important antimicrobial resistance genes *cfr*, *tmexCD3-toprJ1* and *bla*_NDM-1_ were characterized, which showed that those genes were located on diverse genetic elements including Tn*7* transposon, plasmid, SXT/R391 ICEs and SGI1 (Figure 3).

*cfr* gene was located on Tn*7* in strains JZ29 and JZ94, pJZ27-cfr in strain JZ27 and ICE*Pmi*ChnJZ57 in strain JZ57. Strains JZ29 and JZ94 showed high genetic similarity (18 SNPs) and shared the same genetic environment for *cfr*. The *cfr* gene in four strains was flanked by insertion sequence IS*26*. The Tn*7* region in JZ29 chromosome harboured various resistance genes including *cfr* and *bla*_DHA-1_, and was divided into two parts separated by a chromosomal DNA fragment of 112.54 kb (PMI3068-PMI3171 and truncated PMI3172; Figure 3A). We hypothesized that an initial single IS*26* transposition event occurred in JZ29 chromosome in locus PMI3172 and has subsequently generated an inversion between two IS*26* elements in opposite orientation, resulting in the right end of Tn*7* and this 112.54 kb chromosomal DNA fragment being in inverse orientation. The *cfr* gene in strain JZ27 was also located on a Tn*7* in plasmid pJZ27-cfr. pJZ27-cfr was 132,678 bp in size, and showed 100% nucleotide identity to plasmid pHBRJC7 (MK630213) recovered from *P. mirabilis* strain of broiler origin in China (Chen et al., 2022). Compared with pHBRJC7, pJZ27-cfr supplemented IS*26*-*cfr*-*rep*US18 region (Figure 3A). The ICE*Pmi*ChnJZ57 harbouring *cfr* was 110,965 bp is size, and showed 99.99% nucleotide identity to ICE*Pmi*ChnSTP3 (MT449450) in *P. mirabilis* strain from swine in China (He et al., 2021). ICE*Pmi*ChnJZ57 lost *floR* in variable region VRIII and *aphA1* gene in hot spot HS4 compared with ICE*Pmi*ChnSTP3 (Figure 3B). Conjugation experiments indicated that pJZ27-cfr and ICE*Pmi*ChnJZ57 that harboured *cfr* gene could be conjugated into *E. coli* EC600, and ICE*Pmi*ChnJZ57 was chromosomally integrated into the 5*′* end of the *prfC* gene.

*tmexCD3-toprJ1* was located on a novel SXT/R391 ICE designated as ICE*Pmi*ChnJZ26 according to the SXT/R391 nomenclature (Burrus et al., 2006). ICE*Pmi*ChnJZ26 was 105,682 bp in size, and had 61% coverage to *tmexCD3-toprJ1*-harbouring ICE*Pci*Chn180 (CP073356) in *Proteus terrae* subsp. cibarius strain SDQ8C180-2 T from chicken in China (Wang et al., 2021a). *tmexCD3-toprJ1* gene cluster was located in VRIII in both ICE*Pmi*ChnJZ26 and ICE*Pci*Chn180 associated with IS*Cfr1* (Figure 3B). Conjugative transfer of ICE*Pmi*ChnJZ26 failed despite three independent attempts to recover transconjugants carrying ICE*Pmi*ChnJZ26. However, the *tmexCD3-toprJ1*-harbouring ICE was detected in four strains, three of which were not clonally related, indicating the mobility of *tmexCD3-toprJ1*-harbouring ICE.

The *bla*_NDM-1_ in JZ109 was located on the chromosomal SGI1 (Figure 3C), which was 40,275 bp in size and showed 100% nucleotide identity (only one base change) to SGI1-1NDM that was firstly reported in clinical *P. mirabilis* strain PmBJ020-1 (CP065146) from China (Yang et al., 2021). Mobilization assays confirmed that SGI1-1NDM in JZ109 could be successfully transferred to *E. coli* and incorporated into the 3′ end of *trmE* with the help of IncC plasmid pR55.

## Materials and methods

### Sample collection and *Proteus mirabilis* identification

A total of 347 fresh samples (114 chicken meat, 141 pork meat and 92 aquatic products) were purchased from 14 food markets at different locations in Chengdu city, Sichuan province of China from December 2021 to January 2022. The numbers of market stalls selling retail meat and aquatic products were varied from 0 to 20. We collected only one sample from each market stall. The meat samples were stored in sterile bags at 4°C between sampling and further processing. The processing method of *P. mirabilis* identification was modified from the protocol reported previously (Wong et al., 2013). All culture mediums were purchased from Beijing Land Bridge Technology Co., Beijing, China. Briefly, 25 g of meat or aquatic product was placed in 100 ml of buffered peptone water (BPW) and incubated at 37°C for 24 h. Then, the 1 ml BPW-incubated culture was mixed with 9 ml of Rappaport-Vassiliadis *Salmonella* enrichment broth and incubated at 42°C for 24 h. One loopful of broth was streaked onto Mueller Hinton Agar and incubated at 37°C for 16 h to check for the motility of the isolates. The colonies with swarming growth phenotype were streaked on *Shigella* and *Salmonella* agar to obtain single clone. *P. mirabilis* isolates were confirmed by using the BD Phoenix™ 100 Automated Microbiology System (Becton Dickinson, United States). Only one isolate from each sample was selected for further study.

### Genome sequencing, assembly, and bioinformatics analysis

Genomic DNA was extracted using a bacterial genomic DNA extraction kit (Tiangen, China). Whole genomes were sequenced using the Illumina HiSeq platform (150 bp paired-end reads with about 200-fold average coverage), and the clear data were assembled to draft genomes using the software SPAdes v3.15.4. Antimicrobial resistance genes were found using ResFinder 4.1.^1^ SNPs from genomes of the *P. mirabilis* strains were called and a phylogeny based on the concatenated alignment of the high-quality SNPs was inferred by using CSI Phylogeny 1.4^2^ with parameters as defaults. A threshold of 5 SNPs among isolates was considered likely to have an clonal relationship (Dallman et al., 2015; Kanagarajah et al., 2018). The phylogenetic tree was modified in Itol.^3^

### Antimicrobial susceptibility testing

Antimicrobial susceptibility testing was performed using the standard Kirby–Bauer disk diffusion method according to Clinical and Laboratory Standards Institutes (CLSI) guidelines (CLSI, 2020). Susceptibilities to 16 antimicrobial agents were tested including ampicillin (AMP), amoxicillin-clavulanate acid (AMC), cefotaxime (CTX), ceftazidime (CAZ), cefoxitin (FOX), imipenem (IPM), nalidixic acid (NAL), norfloxacin (NOR), ciprofloxacin (CIP), streptomycin (STR), gentamicin (GEN), amikacin (AMK), chloramphenicol (CHL), florfenicol (FFC), fosfomycin (FOS), and trimethoprim-sulfamethoxazole (SXT). *Escherichia coli* ATCC25922 was used as quality control. MDR was defined as resistance to at least one agent in three or more chemical classes of antimicrobials.

### Genetic environments analysis of clinically important antimicrobial resistance genes

The genomes of the *P. mirabilis* strains harbouring *cfr*, *tmexCD3-toprJ1* and *bla*_NDM-1_ were further sequenced using Nanopore MinION using Rapid Sequencing Kit. The complete genome sequences were assembled by Unicycler v0.5.0 using Nanopore sequencing data combined with Illumina sequencing data. Genetic environments of *cfr*, *tmexCD3-toprJ1* and *bla*_NDM-1_ were analyzed using the BLAST program^4^ and Easyfig v2.2.2.^5^

### Conjugation and mobilization assays

Conjugation experiments were performed by using *P. mirabilis* JZ27 (harbouring plasmid pJZ27-cfr), JZ26 (harbouring ICE*Pmi*ChnJZ26), and JZ57 (harbouring ICE*Pmi*ChnJZ57) as the donor strains and rifampin-resistant *E. coli* EC600 as the recipient strain with selection on nutrient agar plates containing 300 mg/l rifampin and 8 mg/l florfenicol (for *cfr*) or 2 mg/l tigecycline (for *tmexCD3-toprJ1*). The positive transconjugants were further determined by detection of antimicrobial resistance profile and the location of ICE*Pmi*ChnJZ26 and ICE*Pmi*ChnJZ57 in *E. coli* with primers LE1/LE4 and RE4/RE1 (McGrath et al., 2006). Mobilization assay for SGI1 harbouring *bla*_NDM-1_ was carried out as previously described (Siebor et al., 2016), using *E. coli* EC600 harboring an IncC plasmid pR55 as the recipient strain. The transconjugants (EC600 carrying SGI1) were further examined for the presence of the *bla*_NDM-1_ and the location of SGI1 in *E. coli* (Girlich et al., 2015; Siebor et al., 2016).

### Nucleotide sequence accession numbers

The genomes of 89 *P. mirabilis* strains reported in this study have been deposited in National Center for Biotechnology Information and registered BioProject number PRJNA847328. The nucleotide sequences of ICE*Pmi*ChnJZ57, Tn*7* in JZ29, pJZ27-cfr, SGI1 in JZ109 and ICE*Pmi*ChnJZ26 have been deposited in GenBank under accession numbers ON553412, ON553413, ON553414, ON553415 and ON630400, respectively.

## Discussion

Food contamination caused by foodborne pathogenic bacteria, such as *Salmonella* and *Campylobacter*, has attracted worldwide attention. However, the prevalence data of the opportunistic pathogen in food products is limited. In this study we focused on *P. mirabilis*, a species frequently associated with nosocomial infection and food poisoning cases. By using NGS, we confirmed high contamination of *P. mirabilis* that harboured various clinically important antimicrobial resistance genes in retail meat and aquatic products.

Our study found that the isolation rate of *P. mirabilis* in retail meat products, especially in chicken meat (isolation rate, 54.39%), was higher than that in aquatic products. This might be due to the fact that the meat products were contaminated by *P. mirabilis* from intestinal tracts of chickens and swines at slaughter. A study from Hong Kong, China reported that 85% (50/58) raw chicken carcass samples were positive for *P. mirabilis* (Wong et al., 2013). Another study from Belgium also reported that *P. mirabilis* was isolated from 29 out of 80 broiler carcasses (36.25%) (Yu et al., 2021). These results indicated that meat products, especially chicken products, were seriously contaminated with *P. mirabilis*. More stringent biosecurity measures are needed in slaughtering, processing and retailing to control t0not only pathogenic bacteria but also opportunistic pathogens.

NGS technology is more and more widely used in tracing the transmission of pathogenic microorganisms. Molecular typing based on whole genome data has higher resolution than traditional PFGE, and can obtain genes information including antimicrobial resistance genes (Boolchandani et al., 2019), which has greater advantage as the cost of NGS decreases. However, genomic data of *P. mirabilis* from food sources is limited. In this study, we provided genomic data of 89 *P. mirabilis* strains, and the phylogenetic analysis showed that the strains had high genetic diversity, indicating that those strains had diverse origin. We also found that the clonal transmission could have occurred in different food markets and different meat products. Given that different meat products in different food markets in Chengdu city might come from the same wholesale meat market, It might be due to the presence of cross contamination of *P. mirabilis* in different meat products from the same wholesale market. Further studies can obtain more genomic data of *P. mirabilis* from animal, food and clinical sources to trace the spread of *P. mirabilis* in the food chain.

Antimicrobial resistance in *P. mirabilis* has become a serious issue for public health (Girlich et al., 2020). The ESBLs and fluoroquinolones resistance genes have been prevalent in *P. mirabilis* (Wong et al., 2013; Huang et al., 2015; Li et al., 2022). In this study, we found that 91.01% strains displayed MDR profiles. Fluoroquinolones and aminoglycosides resistance gene *aac(6′)-Ib-cr*, ESBLs *bla*_CTX-M_ (especially variant *bla*_CTX-M-65_) and fosfomycin resistance gene *fosA3* were prevalent in *P. mirabilis* in retail meat and aquatic products. It is worth noting *P. mirabilis* strains also harboured *cfr*, *tmexCD3-toprJ1* and *bla*_NDM-1_ conferring resistance to the last-resort drugs oxazolidinones, tigecycline and carbapenems, respectively. Diverse genetic elements including Tn*7* transposon, plasmid, SXT/R391 integrative conjugative elements and *Salmonella* genomic island 1, were associated with the horizontal transfer of those resistance genes, which would not only promote the spread of those resistance genes in *P. mirabilis*, but also might spread to Enterobacteriaceae pathogens including *Salmonella*.

## Conclusion

Our study revealed high contamination of *P. mirabilis* harbouring various clinically important antimicrobial resistance genes in retail meat and aquatic products from food markets in China. The last-resort drugs resistance genes were associated with diverse mobile genetic elements, and had potential to spread to Enterobacteriaceae pathogens. More attention should be paid to monitor the presence of MDR opportunistic pathogen in retail meat products.

## Data availability statement

The datasets presented in this study can be found in online repositories. The names of the repository/repositories and accession number(s) can be found in the article/Supplementary material.

## Author contributions

W-QM, Y-YH, and LZ wrote the manuscript and collected the data. Other authors have given some help and suggestions during the experiment and the writing of the manuscript. All authors contributed to the article and approved the submitted version.

## Funding

This work was supported by the National Natural Science Foundation of China (grant nos. 32100147 and U21A20257), Natural Science Foundation of Sichuan Province (2022NSFSC0076) and the Sichuan Science and Technology Program (2021ZDZX0010).

## Conflict of interest

The authors declare that the research was conducted in the absence of any commercial or financial relationships that could be construed as a potential conflict of interest.

## Publisher’s note

All claims expressed in this article are solely those of the authors and do not necessarily represent those of their affiliated organizations, or those of the publisher, the editors and the reviewers. Any product that may be evaluated in this article, or claim that may be made by its manufacturer, is not guaranteed or endorsed by the publisher.

## Footnotes

^1^ https://cge.cbs.dtu.dk/services/ResFinder/

^2^ https://cge.cbs.dtu.dk/services/CSIPhylogeny/

^3^ https://itol.embl.de/

^4^ http://blast.ncbi.nlm.nih.gov/Blast.cgi

^5^ http://mjsull.github.io/Easyfig/
